# Supplementary material for: Metabolome Profiling and Predictive Modeling of Dark Green Leaf Trait in Bunching Onion Varieties
Source: Metabolites. 2025 Mar 26;15(4):226. doi: 10.3390/metabo15040226 (PMC12028952; doi:10.3390/metabo15040226)
Supplement: Supplementary file 1 [file metabolites-15-00226-s001.zip › supplementary Figures.pdf]

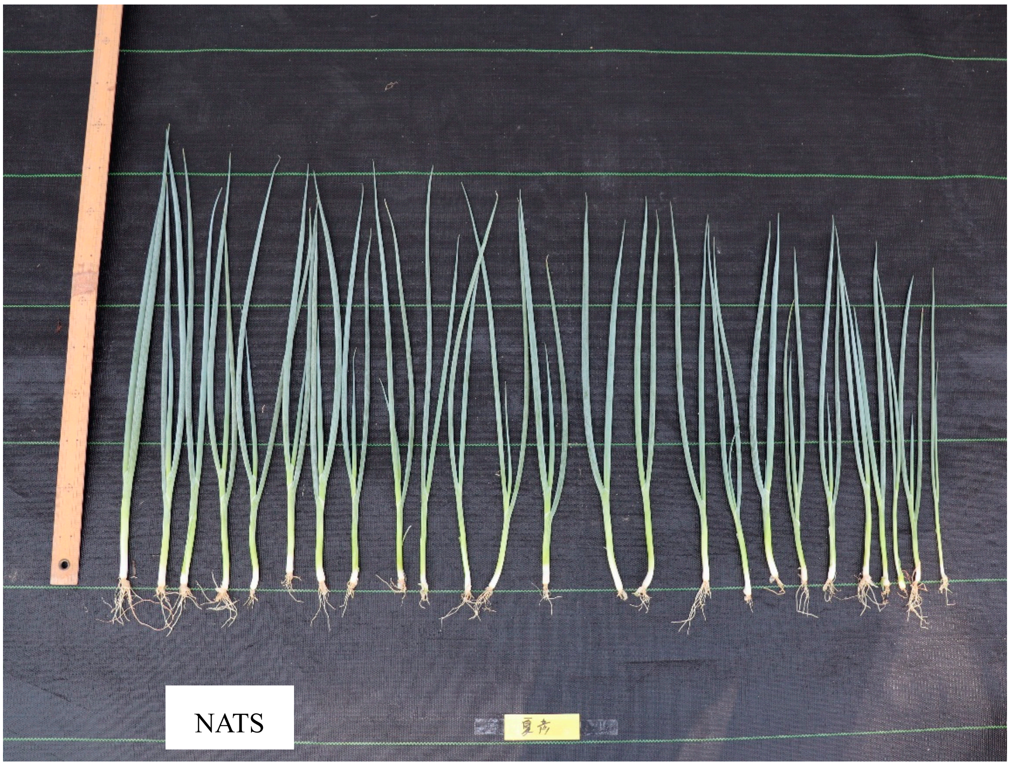

Figure S1. Plants of ‘Nastsuhiko’ (NATS).

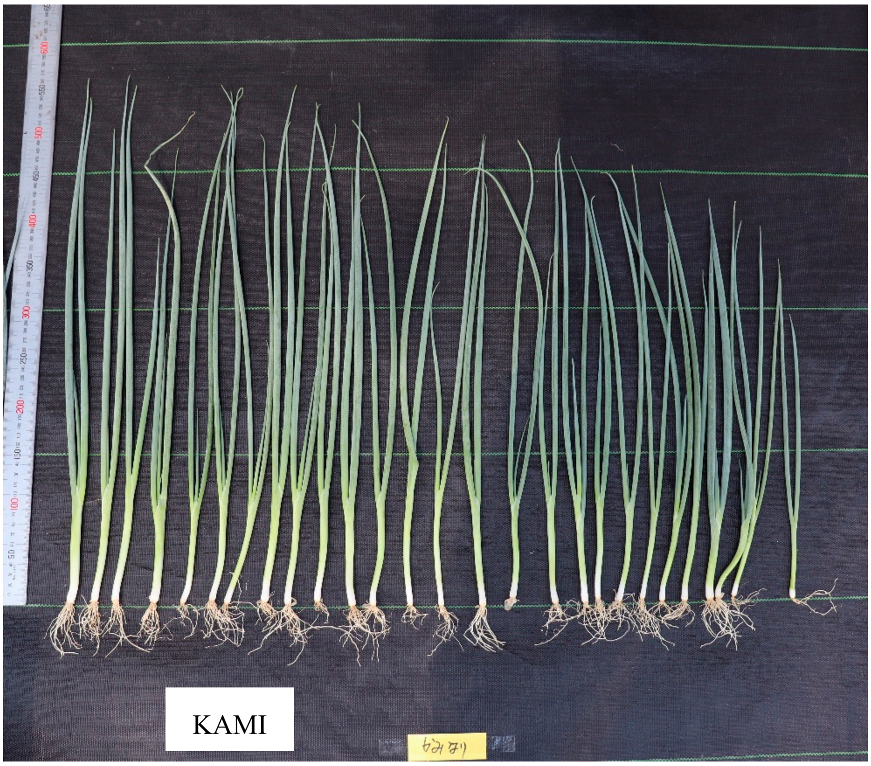

Figure S2. Plants of ‘Kaminari’ (KAMI).

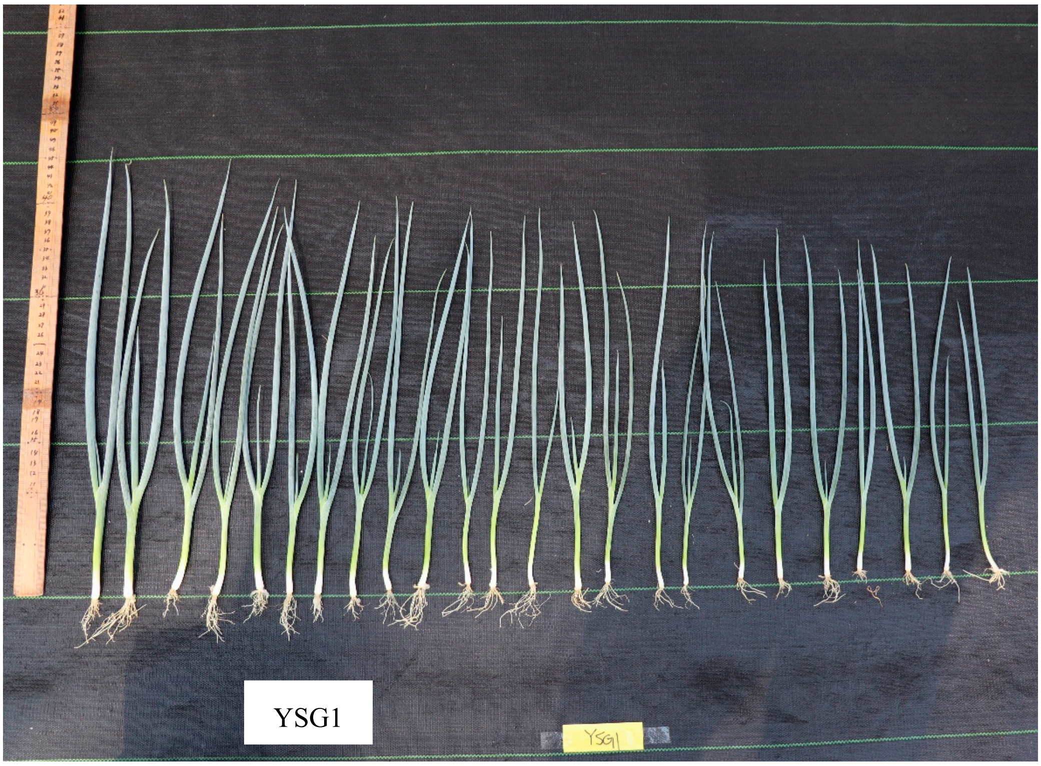

Figure S3. Plants of ‘YSG1go’ (YSG1).

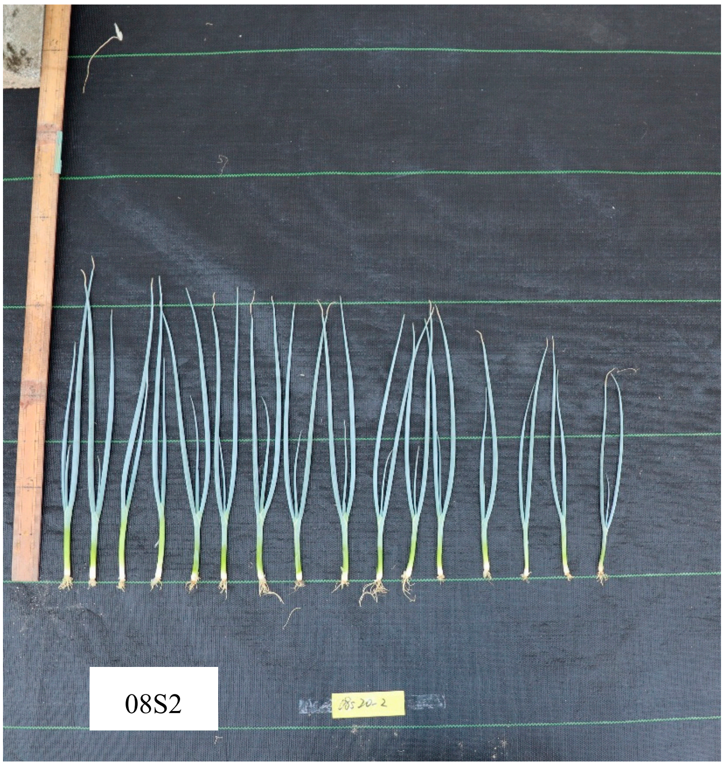

Figure S4. Plants of ‘08S20-2’ (08S2).

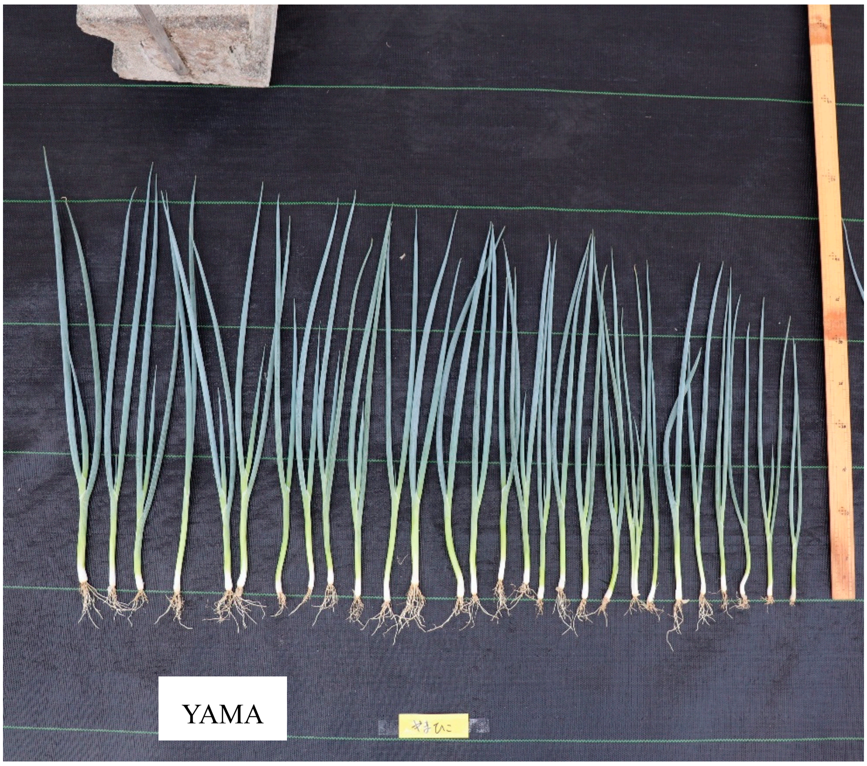

Figure S5. Plants of ‘Yamahiko’ (YAMA).

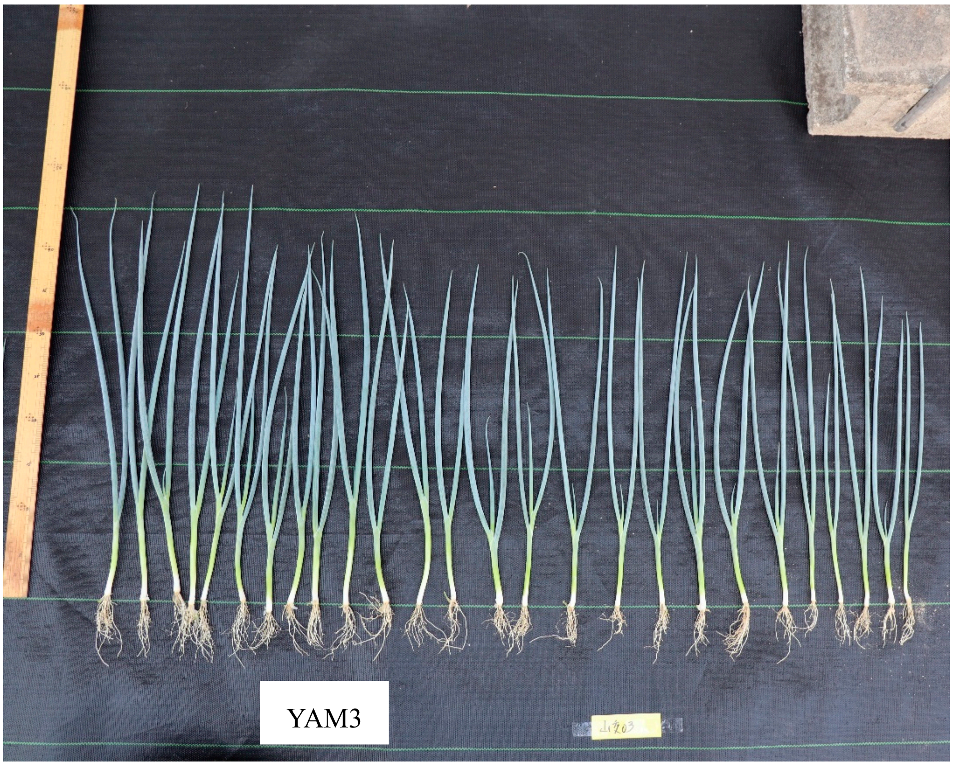

Figure S6. Plants of ‘Yamakou03’ (YAM3).

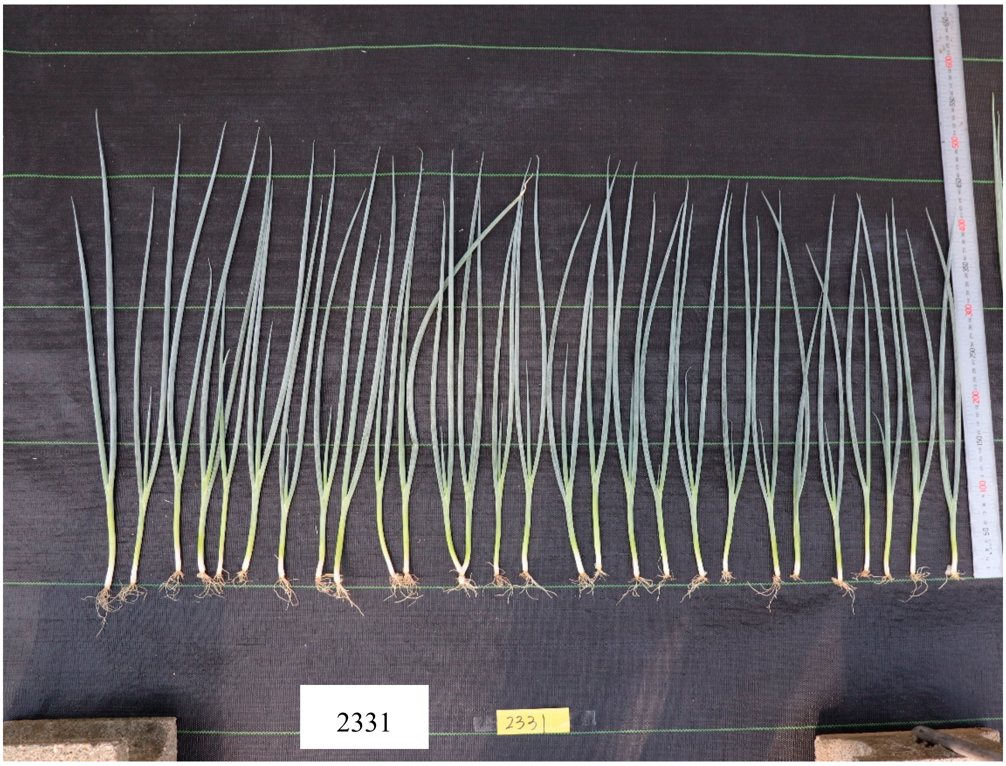

Figure S7. Plants of ‘2331’ (2331).

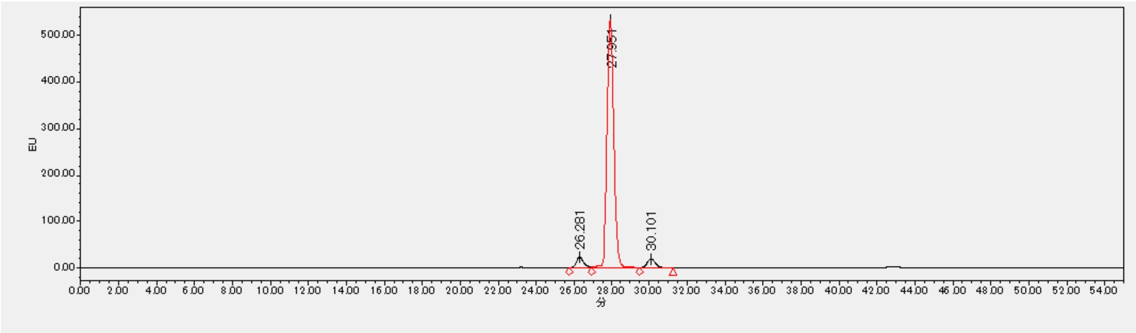

Figure S8. Chromatograms of chlorophyll a obtained using HPLC with a fluorescence detector.

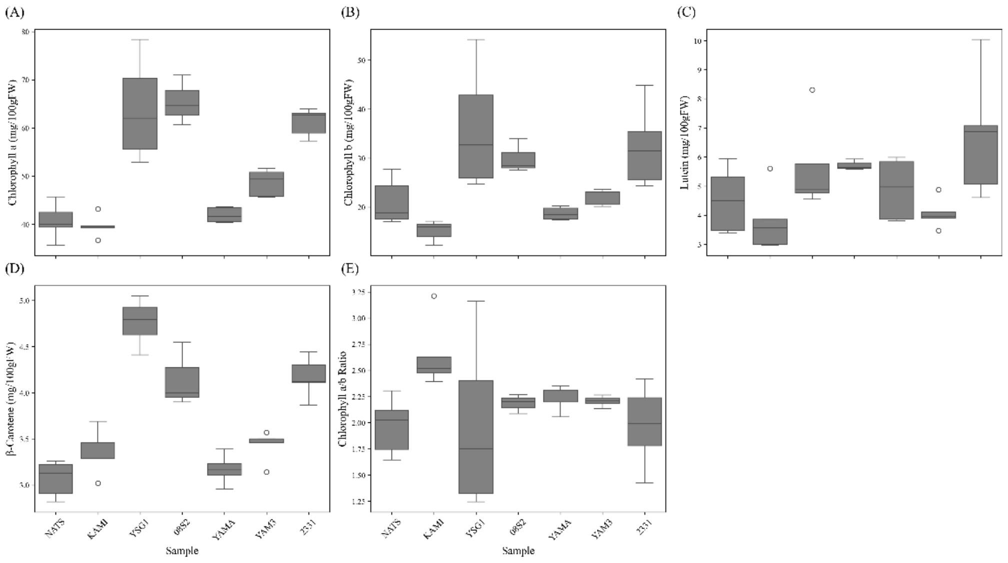

Figure S9. Boxplot of pigment compounds, including chlorophyll *a* (A), chlorophyll *b* (B) lutein (C), *β*-carotene (D), and chlorophyll *a*/*b* ratio (E).

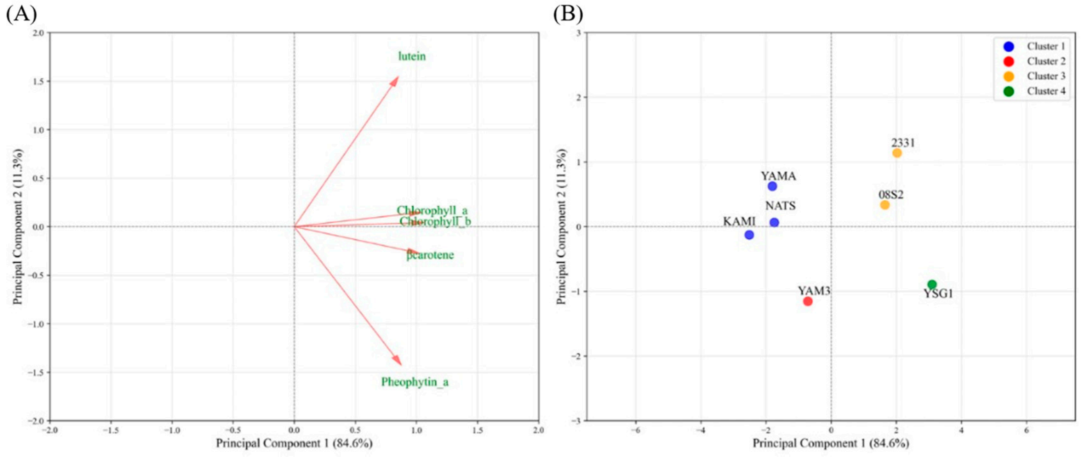

Figure S10. PCA loading plot (A) and PCA score plot with hierarchical clustering (B) of the dataset based on five pigment compounds.
